# Supplementary material for: MetaRibo-Seq measures translation in microbiomes
Source: Nat Commun. 2020 Jun 29;11:3268. doi: 10.1038/s41467-020-17081-z (PMC7324362; doi:10.1038/s41467-020-17081-z)
Supplement: Supplementary file 10 — Supplementary Data 7 [file 41467_2020_17081_MOESM10_ESM.zip › File2/Confidence_High_Taxonomy/49121_out.krona.html]

Javascript must be enabled to view this page.

members
magnitude
magnitudeUnassigned
count
unassigned
taxon
rank

49121\_out

2

2
2
superkingdom

1239
2
phylum

class
2
186801

order
186802
2

family
31979
1

1
1649459
genus

species

SRS021484\_contig\_number\_contig-100\_2560.175727
154046
1

541000
1
family

1
1263
genus


SRS1054691\_contig\_number\_1502
1816688
1
species
